# Materia Medica and Therapeutics

**Published:** 1866-01

**Authors:** 


### MATERIA MEDICA AND THERAPEUTICS.

### *The Hypophosphites—Their Therapeutic Value.*

Under this title, Dr. Ira D. Brown, of Albany, N. Y., contributes to the *Boston Medical and Surgical Journal*, (Dec. 21, 1865,) an interesting paper. After stating the proposition of Dr. Churchill, relative to the importance of phosphorus in the system, Dr. Brown relates a number of cases which exhibited the beneficial effects of the hypophosphites. Used in solution, (calc. hypophos., sod. hypophos. aa℥ij; aquae Oj. M. A tablespoonful to be taken thrice daily,) the effects consisted in an improvement of the digestion, and relief of those nervous disorders which are the consequence of imperfect nutrition. The remedy should be continued for six weeks or two months. Great care should be observed in the selection of the drug, as many worthless imitations are put upon the market.

Few remedies have an *a priori* reputation so good as these preparations, and experience in their use will not disappoint expectations. The acknowledged importance of phosphorus as a constituent of the nervous tissue has rendered desirable an efficient means for its introduction into the system suffering for want of the element. The researches of the physiological chemist proposed the combination in question, simple, unirritating, easy of assimilation, and positively nutritive in quality. Their worth has been thoroughly tested, and may now be considered to be as completely established as that of cod liver oil or quinine. If their properties are accurately propounded, they will be used with intelligence; and, though affording no universal panacea for the ills to which flesh is heir, will prove to be an invaluable addition to the means of restoring nervous tissues.

